# P62/Ubiquitin IHC Expression Correlated with Clinicopathologic Parameters and Outcome in Gastrointestinal Carcinomas

**DOI:** 10.3389/fonc.2015.00070

**Published:** 2015-03-30

**Authors:** Amr Mohamed, Alkhoder Ayman, Johnson Deniece, Tengteng Wang, Charles Kovach, Momin T. Siddiqui, Cynthia Cohen

**Affiliations:** ^1^Department of Pathology and Laboratory Medicine, Emory University School of Medicine, Atlanta, GA, USA; ^2^Department of Medicine, Morehouse School of Medicine, Atlanta, GA, USA; ^3^Department of Epidemiology, Rollins School of Public Health, Emory University, Atlanta, GA, USA

**Keywords:** P62, ubiquitin, immunohistochemical expression, GI carcinoma

## Abstract

P62 and ubiquitin are small regulatory proteins demonstrated to have implications in the prognosis and survival of various malignancies including: hepatocellular, breast, ovarian, and some gastrointestinal carcinomas. Several trials studied the link of their activity to the extrinsic apoptosis pathway and showed that their autophagy modification has a critical stand point in tumorigenesis. These findings explain their vital role in controlling the process of cell death and survival. It has been shown recently that p62 and ubiquitin overexpression in different types of cancers, such as triple negative breast and ovarian cancers, have directly correlated with incidence of distant metastases. We aim to evaluate p62/ubiquitin expression in gastrointestinal carcinomas of gastric, colonic, and pancreatic origin, and correlate with annotated clinicopathologic data. In gastric carcinoma (61), positive p62 nuclear expression was noted in 57% and cytoplasmic in 61%, while positive ubiquitin was nuclear expressed in 68.8%, and cytoplasmic in 29.5%. In colon carcinoma (45), positive p62 nuclear expression was noted in 29% and cytoplasmic in 71%, while positive ubiquitin was nuclear in 58% and cytoplasmic in 44%. In pancreatic cancer (18), positive p62 nuclear expression was noted in 78% and cytoplasmic in 56%, while positive ubiquitin was nuclear in 83% and cytoplasmic in 72%. Normal gastric (6), colon (4), and pancreatic (4) tissues were negative for both P62 and ubiquitin (nuclear and cytoplasmic staining <20%). Ubiquitin high expression was associated with more lymph node metastases in colon (4.14 vs 1.70, *P* = 0.04), and pancreatic adenocarcinomas (3.07 vs 0.33, *P* = 0.03). Also, ubiquitin high expression was associated with worse pancreatic adenocarcinoma overall survival (1.37 vs 2.26 mos, *P* = 0.04). In addition, gastric cancer patients with high p62 expression tend to have more poorly differentiated grade when compared to those with low expression (21 vs 17, *P* = 0.04) but less lymph node metastases (2.77 vs 5.73, *P* = 0.01). P62 and ubiquitin expression did not correlate with other clinicopathologic parameters in gastric, colon or pancreatic denocarcinomas. The results suggest that p62 and ubiquitin are highly expressed in gastric, colonic, and pancreatic carcinomas. High ubiquitin expression was noted to have an impact on number of lymph node metastases in patients with colon and pancreatic adenocarcinomas, but on overall survival only in patients with pancreatic adenocarcinoma. Also, P62 high expression is correlated with poor differentiation, but less lymph node metastases, in gastric carcinoma.

## Introduction

Ubiquitin is a small regulatory protein that was discovered in 1975 by Goldstein (1). It has been found in almost all eukaryotic cells. It consists of 76 amino acids and is encoded by four different genes in the human genome: UBB, UBC, UBA52, and RPS27A. Critical cellular processes are regulated, in part, by maintaining the appropriate intracellular levels of proteins through a balance between protein synthesis and degradation. The ubiquitin–proteasome pathway is a major pathway for the targeted degradation of proteins. This pathway involves multi-step enzymatic reactions catalyzed by a cascade of enzymes that include: ubiquitin-activating enzyme E1, ubiquitin-conjugating enzyme E2, and ubiquitin ligase E3 (2). In fact, data shows that ubiquitin plays a vital role in protein degradation and in many other cellular functions such as: cell growth, cell cycle regulation, immune system functionality, and DNA repair processes (3). In addition, it is engaged in the regulation of turnover as well as the activity of many target proteins involved in cell proliferation, differentiation, and cell death (4). Therefore, any disruption in its pathway is linked to several diseases including: neurodegenerative diseases, genetic disorders, cancers, and other immunological disorders.

P62 is an ubiquitin binding protein which induces activation of multiple upstream signaling pathways, including those triggered by epidermal growth factor (EGF) receptors. In several studies, immunohistochemical staining of p62 has been shown in different parts of the gastrointestinal tract (stomach, esophagus, large intestine) including cancers (5, 6). Its expression has been associated with cell differentiation and tumor metastasis in different types of malignant tumors (such as breast cancer) (7). Many trials suggest that this expression of p62 in tissues, and the appearance of autoantibody to p62, might be related to manifestations of malignancy (6). The accumulation of p62 is reported to be associated with a higher risk of distant metastases, poorer prognosis (particularly in breast cancer), and to be predictive of response to clinical treatments (5).

Moreover, regulation of ubiquitin is involved in tumor progression and oncogenesis. Its overexpression in human cancers correlates with chemoresistance and poor clinical prognosis (2). This critical role of ubiquitin in protein turnover in cell cycle regulation makes this process a target for oncogenic mutations and codes for several oncogenes in different malignancies (8, 9) such as colonic and renal cell carcinoma.

In gastrointestinal (GI) carcinomas, there are few studies of the expression of P62/ubiquitin. It has been shown that ubiquitin expression in pancreatic adenocarcinoma significantly correlates with clinical stage, degree of histologic differentiation, lymph node metastasis, and poor overall survival (10). However, there are little data about the role of both p62 and ubiquitin in gastric and colorectal carcinogenesis, prognosis, and aspects of its inhibition for therapeutic purposes.

In this study, in order to understand the roles p62/ubiquitin play in gastrointestinal carcinomas of gastric, colorectal, and pancreatic origin, we carried out immunohistochemical analyses of p62/ubiquitin expression in a cohort of patients with annotated clinicopathologic data. Expression of both markers was correlated with clinicopathologic parameters and survival.

## Design

### Study group

The study group was composed of 61 gastric, 45 colon, and 18 pancreatic adenocarcinomas from patients diagnosed at Emory University Hospital between 2000 and 2013 with tissue available in tissue microarrays (TMAs). TMAs were constructed using two 1.0 mm tissue cores from each neoplasm, and included non-neoplastic tissues. Permission to use the TMAs and to review pathology reports and patient charts was obtained from the Institutional Review Board of Emory University.

### Immunohistochemistry

Five micron sections of the formalin-fixed, paraffin-embedded TMAs were tested for the presence of p62 using mouse monoclonal antibody (SQSTM1) (Abcam, Cambridge, MA, USA) (dilution 1/3200); and ubiquitin using rabbit antihuman polyclonal antibody (Dako Corp., Carpintaria, CA, USA) (dilution 1/800). Sections were deparaffinized in xylene and grades of alcohol, then rehydrated in water. Antigen retrieval was performed in citrate buffer (pH 6.0) using an electric pressure cooker for 3 min at 12–15 pounds per square inch (120°C), and cooled for 10 min prior to immunostaining. All slides were loaded on an automated system (Dako Autostainer) and exposed to 3% H_2_O_2_ for 5 min. Tissues were then exposed to primary antibody for 30 min. Envision plus (Dako) detection system was used, with labeled polymer-horseradish peroxidase for 30 min, diaminobenzidine as chromogen for 5 min, and DAKO automation hematoxylin as counterstain for 15 min.

These incubations were performed at room temperature; between incubations, sections were washed with Tris-buffered saline. Cover slipping was performed using the Tissue Tek SCA (Sakura Finetek USA, Torrance, CA, USA) automatic cover slipper. Positive controls were hepatocellular carcinoma known to be positive for p62 and ubiquitin. Negative controls had primary antibody replaced by buffer.

P62 and ubiquitin expression was nuclear and cytoplasmic. Intensity was categorized as negative (0), low (1), moderate (2), and high (3). A staining area of >20% of tumor cells was used as a cutoff for positivity. The Q-Score, generated by multiplying the staining intensity by the percent of tumor cell positivity, was divided into negative (0), low (<100), moderate (100–200), and high (200–300). Normal gastric, colon, and pancreatic tissues were included as negative controls.

### Statistics

After categorizing the subjects into positive and negative groups based on the 20% cutoff of staining area, the staging of these three types of cancer was classified into early (stage 0–3A) and advanced stage (equal or more than stage 3B). The overall survival time of all the three types of cancer were not normally distributed and were log-transformed for the following analyses. Chi-square or Fisher’s exact test was used for assessing the comparability of cancer staging (pathologic stage, tumor size, lymph node metastases, and distant metastases) between positive and negative groups. A two-sample *t*-test was used to evaluate the association between log-transformed survival time and the positivity of tumor cells. All the statistical analyses were conducted using SAS statistical software version 9.3 (SAS Institute Inc., Cary, NC, USA), and *P* < 0.05 was considered statistically significant.

## Results

**Figure 1:**
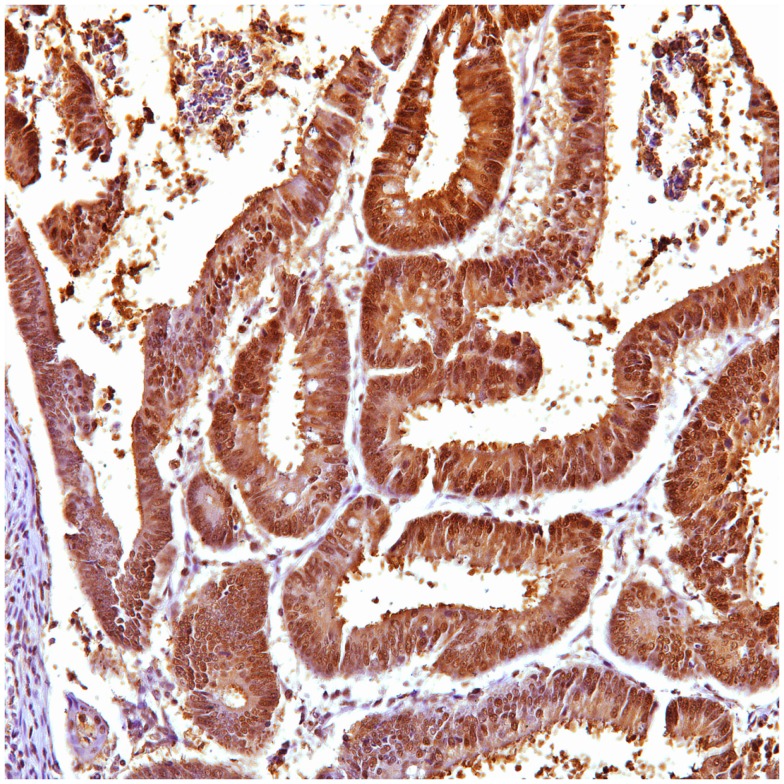
**Ubiquitin cytoplasmic positive in gastric carcinoma**.

**Figure 2:**
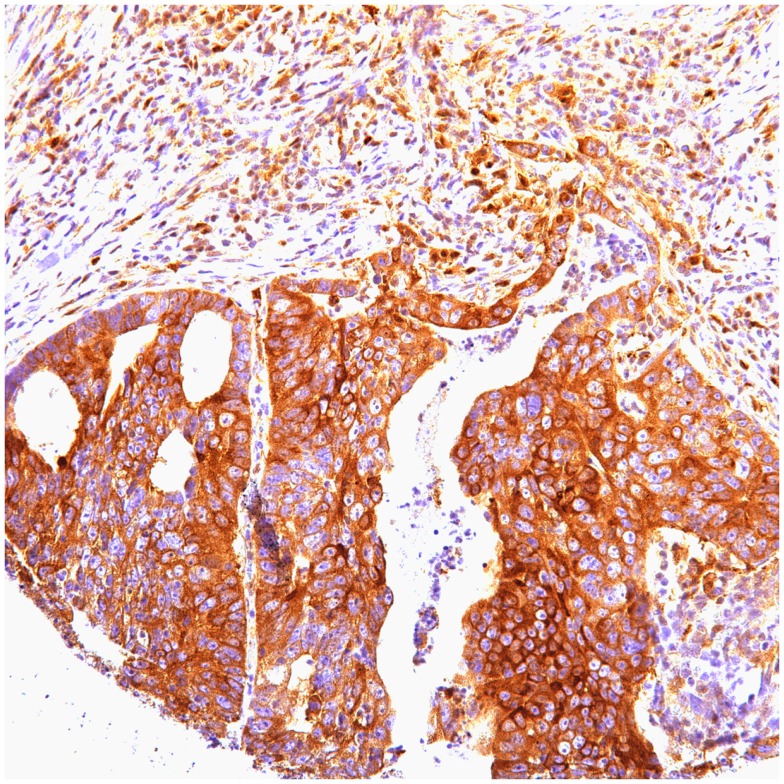
**Cytoplasmic positive in gastric carcinoma**.

**Figure 3:**
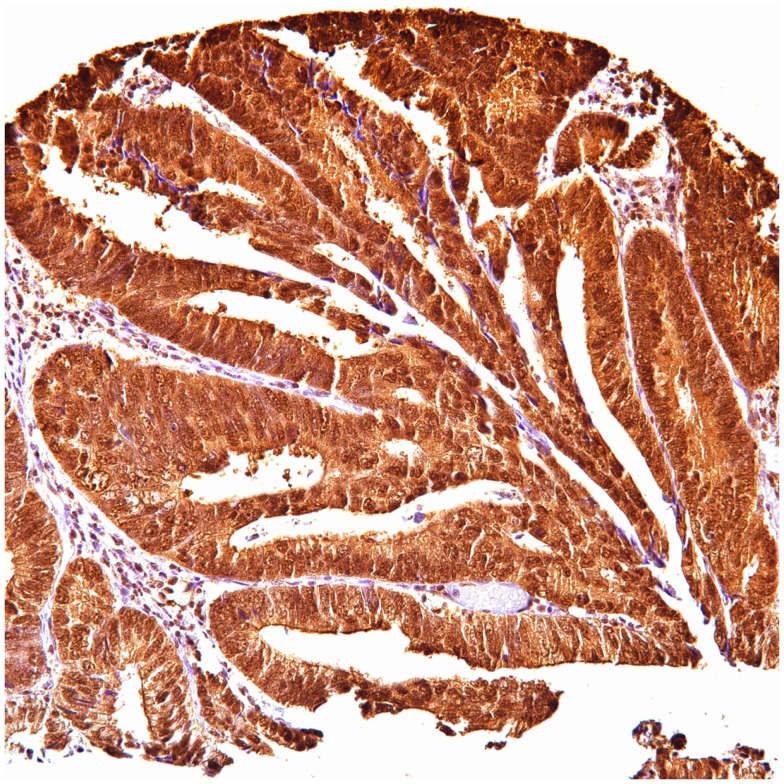
**Ubiquitin cytoplasmic positive in colon carcinoma**.

**Figure 4:**
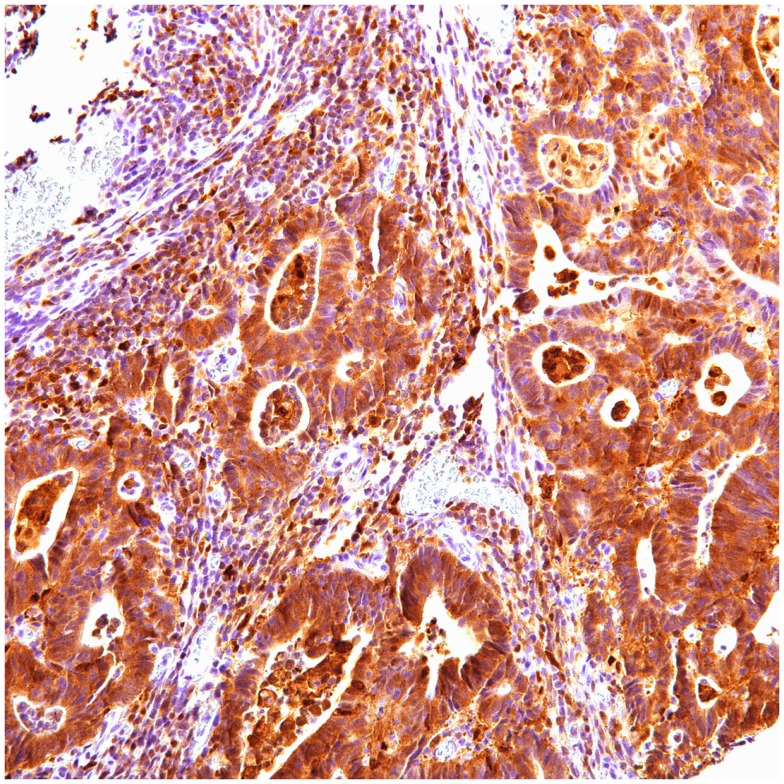
**P62 cytoplasmic positive in colon carcinoma**.

Of 124 cases, there were 61 gastric, 45 colorectal, and 18 pancreatic carcinoma. Tables 1–3 show the immunohistochemistry profile for P62 and ubiquitin respectively in the above carcinomas (Figures 1–4).

**Table 1:** **Analysis of tumor variables and overall survival in patients with gastric cancer (*n* = 61)**.

Characteristic	Positive	Negative	*P*-value*
Age (median/range): (64/53–84)			
Gender			
Males, *n* (%): 42 (69%)			
Females, *n* (%): 19 (31%)			
**Ubiquitin nucleus**			
Total number (%)	46/61 (68.6%)	15/61 (24.5%)	
Grade			0.81
I *n* (%)	0 (0%)	1 (6.6%)	
II *n* (%)	12 (26%)	8 (53.3%)	
III *n* (%)	34 (74%)	6 (40.1%)	
Tumor size (cm)^a^, mean ± SD	1.47 ± 0.70	1.30 ± 0.67	0.42
LNS_number, mean ± SD	3.39 ± 4.68	6.00 ± 4.00	0.06
Distant mets, *n* (%)			0.89
Yes	10 (21.74)	3 (20.00)	
No	36 (78.26)	12 (80.00)	
Stage, *n* (%)			0.65
Early	27 (72.97)	10 (27.03)	
Advanced	18 (78.26)	5 (21.74)	
Survival time (months)^a^, mean ± SD	2.41 ± 1.13	2.66 ± 1.42	0.53
**Ubiquitin cytoplasm**			
Total number (%)	18/61 (30)	43/61 (70)	
Grade			0.09
I *n* (%)	0 (0%)	1 (1.6%)	
II *n* (%)	6 (33.3%)	16 (37.2%)	
III *n* (%)	12 (66.7)	26 (61.2%)	
Tumor size (cm)^a^, mean ± SD	1.52 ± 0.61	1.39 ± 0.73	0.49
LNS_number, mean ± SD	4.33 ± 6.17	3.90 ± 3.90	0.75
Distant mets, *n* (%)			0.14
Yes	6 (33.33)	7 (16.28)	
No	12 (66.67)	36 (83.72)	
Stage, *n* (%)			0.37
Early	12 (32.43)	25 (67.57)	
Advanced	5 (21.74)	18 (78.26)	
Survival time (months)^a^, mean ± SD	2.68 ± 0.91	2.38 ± 1.30	0.32
**P62 nucleus**			
Total number (%)	35/61 (57)	26/61 (43)	
Grade			0.04
I *n* (%)	1 (2.8)	0 (0)	
II *n* (%)	13 (37.2)	9 (34.7)	
III *n* (%)	21 (60)	17 (65.3)	
Tumor size (cm)^a^, mean ± SD	1.46 ± 0.74	1.39 ± 0.62	0.72
LNS_number, mean ± SD	2.77 ± 3.87	5.73 ± 5.08	0.01
Distant mets, *n* (%)			0.36
Yes	6 (17.14)	7 (26.92)	
No	29 (82.86)	19 (73.08)	
Stage, *n* (%)			0.99
Early	21 (56.76)	16 (43.24)	
Advanced	13 (56.52)	10 (43.38)	
Survival time (months)^a^, mean ± SD	2.37 ± 0.19	2.60 ± 1.33	0.48
**P62 cytoplasm**			
Total number (%)	37/61 (61)	24/61 (39)	
Grade			0.44
I *n* (%)	0 (0)	1 (4)	
II *n* (%)	14 (37.8)	8 (33.5)	
III *n* (%)	23 (62.2%)	15 (62.5)	
Tumor size (cm)^a^, mean ± SD	1.35 ± 0.71	1.55 ± 0.65	0.26
LNS_number, mean ± SD	4.28 ± 4.36	3.68 ± 5.07	0.62
Distant mets, *n* (%)			0.09
Yes	5 (13.89)	8 (32.00)	
No	31 (86.11)	17 (68.00)	
Stage, *n* (%)			0.52
Early	21 (56.76)	16 (43.24)	
Advanced	15 (65.22)	8 (34.78)	
Survival time (months)^a^, mean ± SD	2.47 ± 1.10	2.46 ± 1.36	0.97

*P-values derived from chi-square or Fisher’s exact test for stage categories and two-sample *t*-test for log-transformed survival time.

*^a^Log-transformed*.

**Table 2:** **Multivariate analysis of tumor variables and overall survival in patients with colon cancer (*n* = 45)**.

Characteristic	Positive, n (%)	Negative, n (%)	*P*-value*
Age (median/range): (60.6/50–81)			
Gender			
Males, n (%): 28 (62%)			
Females, n (%): 17 (38%)			
**Ubiquitin nucleus**			
Total number (%)	26/45 (58)	19/45 (42)	
Grade			0.35
I *n* (%)	0 (0)	1 (5.2)	
II *n* (%)	20 (77)	16 (84.2)	
III *n* (%)	6 (23)	2 (10.5)	
Tumor size (cm), mean ± SD	4.68 ± 2.45	4.67 ± 2.09	0.99
LNS_number, mean ± SD	2.83 ± 3.96	3.00 ± 4.41	0.90
Distant mets, n (%)			0.07
Yes	5 (16.67)	7 (46.67)	
No	25 (83.33)	8 (53.33)	
Stage, *n* (%)			0.14
Early	15 (78.95)	4 (21.05)	
Advanced	15 (57.69)	11 (42.31)	
Survival time (months)^a^, mean ± SD	2.67 ± 1.67	2.62 ± 1.77	0.94
**Ubiquitin cytoplasm**			
Total number (%)	20/45 (44)	25/45 (56)	
Grade			0.56
I *n* (%)	0 (0)	1 (4)	
II *n* (%)	16 (80)	21 (84)	
III *n* (%)	4 (20)	3 (12)	
Tumor size (cm), mean ± SD	4.98 ± 2.59	4.39 ± 2.03	0.40
LNS_number, mean ± SD	4.14 ± 1.85	1.70 ± 0.77	0.04
Distant mets, *n* (%)			0.44
Yes	7 (31.82)	5 (21.74)	
No	15 (68.18)	18 (78.26)	
Stage, *n* (%)			0.86
Early	9 (47.37)	10 (52.63)	
Advanced	13 (50.00)	13 (50.00)	
Survival time (months)^a^, mean ± SD	2.40 ± 1.59	2.90 ± 1.77	0.34
**P62 nucleus**			
Total number (%)	13/45 (29)	32/35 (71)	
Grade			0.06
I *n* (%)	0 (0)	1 (3)	
II *n* (%)	11 (84.6)	24 (75)	
III *n* (%)	2 (15.4)	7 (22)	
Tumor size (cm), mean ± SD	4.59 ± 2.37	4.72 ± 2.32	0.87
LNS_number, mean ± SD	4.27 ± 5.28	2.20 ± 3.18	0.11
Distant mets, *n* (%)			0.07
Yes	1 (6.67)	11 (36.67)	
No	14 (93.33)	19 (63.33)	
Stage, *n* (%)			0.39
Early	5 (26.32)	14 (73.68)	
Advanced	10 (38.46)	16 (61.54)	
Survival time (months)^a^, mean ± SD	2.45 ± 1.66	2.76 ± 1.71	0.57
**P62 cytoplasm**			
Total number (%)	32/45 (71)	13/35 (29)	
Grade			0.15
I *n* (%)	1 (3)	0 (0)	
II *n* (%)	25 (78)	12 (92)	
III *n* (%)	6 (19)	1 (8)	
Tumor size (cm), mean ± SD	4.67 ± 2.06	4.71 ± 3.17	0.96
LNS_number, mean ± SD	3.09 ± 4.38	2.20 ± 2.74	0.55
Distant mets, *n* (%)			0.28
Yes	8 (22.86)		4 (40.00)
No	27 (77.14)		6 (60.00)
Stage, *n* (%)			0.87
Early	15 (78.95)	4 (21.05)	
Advanced	20 (76.92)	6 (23.08)	
Survival time (months)^a^, mean ± SD	2.66 ± 1.59	2.63 ± 2.05	0.97

*P-values derived from chi-square or Fisher’s exact test for stage categories and two-sample *t*-test for log-transformed survival time.

*^a^Log-transformed*.

**Table 3:** **Multivariate analysis of tumor variables and overall survival in patients with pancreatic adenocarcinoma (*n* = 18)**.

Characteristic	Positive, *n* (%)	Negative, *n* (%)	*P*-value*
Age (median/range): 64 (50–75)			
Gender			
Males, *n* (%): 10 (66.6%)			
Females, *n* (%): 5 (33.3%)			
**Ubiquitin nucleus**			
Total number (%)	15/18 (83)	3/18 (17)	
Grade			0.22
I *n* (%)	3 (20)	0 (0)	
II *n* (%)	8 (53)	0 (0)	
III *n* (%)	4 (27)	0 (0)	
Tumor size (cm)^a^, mean ± SD	1.57 ± 1.08	1.93 ± 0.72	0.58
LNS (*N*, mean ± SD)	3.07 ± 4.10	0.33 ± 0.58	0.03
Distant mets, *n* (%)			1.00
Yes	1 (6.67)	0 (0.00)	
No	14 (93.33)	2 (100.00)	
Stage, *n* (%)			1.00
Early	14 (87.50)	2 (12.50)	
Advanced	1 (100.00)	0 (0.00)	
Overall Survival (months)^a^, mean ± SD	1.57 ± 1.08	1.93 ± 0.72	0.51
**Ubiquitin cytoplasm**			
Total number (%)	13/18 (72)	5/18 (28)	
Grade			0.77
I *n* (%)	2 (24)	1 (33.3)	
II *n* (%)	7 (58)	1 (33.3)	
III *n* (%)	3 (25)	1 (33.3)	
Tumor size (cm)^a^, mean ± SD	1.14 ± 0.32	1.59 ± 0.73	0.08
LNS_number, mean ± SD	2.77 ± 3.61	2.20 ± 4.92	0.79
Distant mets, *n* (%)			0.61
Yes	0 (0.00)	1 (20.00)	
No	13 (100.00)	4 (80.00)	
Stage, *n* (%)			0.29
Early	12 (75.00)	4 (25.00)	
Advanced	0 (0.00)	1 (100.00)	
Survival time (months)^a^, mean ± SD	1.37 ± 1.08	2.26 ± 0.51	0.04
**P62 nucleus**			
Total number (%)	14/18 (78)	4/18 (22)	
Grade			0.98
I n (%)	3 (23%)	0 (0%)	
II n (%)	6 (46%)	2 (100%)	
III n (%)	4 (30.7)	0 (0%)	
Tumor size (cm)^a^, mean ± SD	1.25 ± 0.54	1.28 ± 0.29	0.92
LNS_number, mean ± SD	2.28 ± 3.65	3.75 ± 4.99	0.52
Distant mets, *n* (%)			1.00
Yes	1 (7.14)	0 (0.00)	
No	13 (92.86)	4 (100.00)	
Stage, *n* (%)			1.00
Early	13 (81.25)	3 (18.75)	
Advanced	1 (100.00)	0 (0.00)	
Survival time (months)^a^, mean ± SD	1.72 ± 1.04	1.34 ± 1.02	0.55
**P62 cytoplasm**			
Total number (%)	10/18 (56)	8/18 (44)	
Grade			0.31
I *n* (%)	2 (22)	1 (16.6)	
II *n* (%)	4 (44)	4 (66.6)	
III *n* (%)	3 (33)	1 (16.6)	
Tumor size (cm)^a^, mean ± SD	1.00 ± 0.37	1.52 ± 0.47	0.02
LNS_number, mean ± SD	2.00 ± 3.61	3.22 ± 4.24	0.51
Distant mets, *n* (%)			1.00
Yes	0 (0.00)	1 (11.11)	
No	9 (100.00)	8 (88.89)	
Stage, *n* (%)			1.00
Early	8 (50.00)	8 (50.00)	
Advanced	0 (0.00)	1 (100.00)	
Survival time (months)^a^, mean ± SD	1.47 ± 1.06	1.77 ± 1.02	0.56

*P-values derived from chi-square or Fisher’s exact test for stage categories and two-sample *t*-test for log-transformed survival time.

*^a^Log-transformed*.

In gastric carcinoma (61), immunohistochemical staining revealed that positive p62 expression was noted [nucleus: 35 (57%) and cytoplasmic 37 (61%)]. P62 nuclear stain was mostly of moderate to high intensity (2–3), cytoplasmic stain mostly of low to moderate intensity (1–2). Positive ubiquitin was present [nucleus: 46 (68.6%) and cytoplasmic 18 (30%)]. Ubiquitin nuclear stain was mostly of moderate to high intensity (2–3), cytoplasmic stain mostly of low intensity (1).

In colon carcinoma (45), positive p62 expression was noted [nucleus:13 (29%) and cytoplasmic 32 (71%)]. P62 nuclear and cytoplasmic stain was mostly of moderate to high intensity (2–3). Positive ubiquitin was present [nucleus: 26 (58%) and cytoplasmic 20 (44%)], with nuclear stain mostly of moderate to high intensity (2–3), and cytoplasmic stain mostly of low intensity (1).

In pancreatic carcinoma (18), positive p62 nuclear expression was noted in 78% (14/18) and cytoplasmic in 56% (10/18). P62 nuclear stain mostly of high intensity (3), cytoplasmic stain mostly of moderate to high intensity (2–3). Positive ubiquitin was nuclear, expressed in 83% (15/18) and cytoplasmic in 72% (13/18). Ubiquitin nuclear and cytoplasmic stain was mostly of high intensity (3).

Normal non-neoplastic gastric (6), colon (4), and pancreatic (4) tissues were negative for both P62 and ubiquitin, nuclear, and cytoplasmic staining (0–1 intensity, <20%).

Ubiquitin high expression was associated with more lymph node metastases in colon (4.14 vs 1.70, *P* = 0.04), and pancreatic adenocarcinomas (3.07 vs 0.33, *P* = 0.03). Also, Ubiquitin high expression was associated with worse pancreatic adenocarcinoma overall survival (1.37 vs 2.26 mos, *P* = 0.04). In addition, gastric cancers with high p62 expression were more often poorly differentiated (21 vs 17, *P* = 0.04), but less lymph node metastases (2.77 vs 5.73, *P* = 0.01) when compared to those with low expression. P62 and ubiquitin expression did not correlate with other clinicopathologic parameters in gastric, colon, or pancreatic adenocarcinomas.

## Discussion

It has been described before that carcinogenesis is a multi-step process involving many complex factors (11). Furthermore, genetic mutation in oncogenes, tumor-suppressor genes, and other tumorigenic factors play an important role in this process. Recently, there has been growing evidence that a number of intracellular proteins and auto-antibodies are associated with increased risk of cancer (12). P62 and ubiquitin are considered autoantibodies that are found in many malignant tumors (6). Previous studies have demonstrated accumulation of p62/Ubiquitin as cytoplasmic autoantigens in sera from patients with different types of cancers (13). Their expression has been reported in the carcinogenesis process (14). Although reported in some cancers such as hepatocellular carcinoma, information regarding their expression in other GI tumors is not studied well. Even now, the association of these regulatory proteins with the behavior of GI carcinomas is still not fully understood.

In the current study, the diversity of p62/Ubiquitin expression was characterized in patients with different digestive system cancers. Our data show that p62/ubiquitin are highly expressed in these GI cancers, and may indicate their role in increased cellular proliferation and promotion of tumorigenesis. However, it is becoming apparent that p62/ubiquitin influences various cellular processes including cell growth, survival and mitosis; their role in promoting carcinogenesis is still a gray area. They exhibit an oncofetal behavior with increased cellular proliferation and malignant transformation (15). Both p62/ubiquitin have a posttranscriptional role modulating the stability and function of certain important mRNAs. They have emerged as crucial molecules in regulation of autophagy, and as modulators of mitotic transit and genomic stability allowing tumor cells to survive under conditions of autophagy defects. In addition, recent studies demonstrated an unanticipated role for p62 in activation of the mammalian target of rapamycin (mTOR) pathway; which is a central regulator of cell growth and autophagy, and is aberrantly activated in many types of cancer such as hepatocellular carcinoma (14, 16). Also, p62 accumulation has been directly correlated with higher risk of distant metastasis in triple negative breast carcinoma patients (5).

Our results confirm the recent growing evidence that a number of intracellular proteins with RNA-binding motifs have been identified in human cancers. High ubiquitin expression is associated with increased frequency of lymph node metastases in both colon and pancreatic adenocarcinomas, and has an impact on overall survival in patients with pancreatic adenocarcinoma being associated with worse overall survival (1.37 vs 2.26 mos, *P* = 0.04).

High p62 expression is associated with poorly differentiation but less lymph node metastases in gastric carcinoma. Our study results do not show correlation between P62 and ubiquitin expression and other clinicopathologic parameters in gastric, colon, and pancreatic adenocarcinomas. In previous studies, p62/ubiquitin overexpression has been associated with increased incidence of recurrence after treatment, and decreased overall survival (2, 5). Our data show that both markers may represent potentially promising therapeutic targets.

In summary, in our current study we extended immunohistochemical analysis from hepatic to other gastrointestinal carcinomas. There was significant expression of p62/ubiquitin in gastric, pancreatic, and colonic carcinomas. In addition, ubiquitin expression seems to have an impact on frequency of lymph node metastases in colon and pancreatic carcinomas and overall survival in patients with pancreatic adenocarcinoma.

## Conflict of Interest Statement

The authors declare that the research was conducted in the absence of any commercial or financial relationships that could be construed as a potential conflict of interest.

## References

[1] Goldstein G Scheid M Hammerling U Schlesinger DH Niall HD Boyse EA. Isolation of a polypeptide that has lymphocyte-differentiating properties and is probably represented universally in living cells. Proc Natl Acad Sci U S A (1975) 72(1):11–5. 10.1073/pnas.72.1.11 1078892 PMC432229

[2] Sun Y. E3 ubiquitin ligases as cancer targets and biomarkers. Neoplasia (2006) 8(8):645–54. 10.1593/neo.06376 16925947 PMC1601942

[3] Bonifacino JS Weissman AM. Ubiquitin and the control of protein fate in the secretory and endocytic pathways. Annu Rev Cell Dev Biol (1998) 14:19–57. 10.1146/annurev.cellbio.14.1.19 9891777 PMC4781171

[4] Genini D Carbone GM Catapano CV. Multiple interactions between peroxisome proliferators-activated receptors and the ubiquitin-proteasome system and implications for cancer pathogenesis. PPAR Res (2008) 2008:195065. 10.1155/2008/195065 18551186 PMC2423003

[5] Luo RZ Yuan ZY Li M Xi SY Fu J He J. Accumulation of p62 is associated with poor prognosis in patients with triple-negative breast cancer. Onco Targets Ther (2013) 6:883–8. 10.2147/OTT.S46222 23888115 PMC3722135

[6] Su Y Qian H Zhang J Wang S Shi P Peng X. The diversity expression of p62 in digestive system cancers. Clin Immunol (2005) 116(2):118–23. 10.1016/j.clim.2005.04.004 15886058

[7] Rolland P Madjd Z Durrant L Ellis IO Layfield R Spendlove I. The ubiquitin-binding protein p62 is expressed in breast cancers showing features of aggressive disease. Endocr Relat Cancer (2007) 14(1):73–80. 10.1677/erc.1.01312 17395976

[8] Mani A Gelmann EP. The ubiquitin-proteasome pathway and its role in cancer. J Clin Oncol (2005) 23(21):4776–89. 10.1200/JCO.2005.05.081 16034054

[9] Chen C Seth AK Aplin AE. Genetic and expression aberrations of E3 ubiquitin ligases in human breast cancer. Mol Cancer Res (2006) 4(10):695–707. 10.1158/1541-7786.MCR-06-0182 17050664

[10] Jiang X Hao HX Growney JD Woolfenden S Bottiglio C Ng N Inactivating mutations of RNF43 confer Wnt dependency in pancreatic ductal adenocarcinoma. Proc Natl Acad Sci U S A (2013) 110(31):12649–54. 10.1073/pnas.1307218110 23847203 PMC3732970

[11] Klaunig JE Kamendulis LM Hocevar BA Oxidative stress and oxidative damage in carcinogenesis. Toxicol Pathol (2010) 38(1):96–109 10.1177/0192623309356453 20019356

[12] Casiano CA Mediavilla-Varela M Tan EM. Tumor-associated antigen arrays for the serological diagnosis of cancer. Mol Cell Proteomics (2006) 5(10):1745–59. 10.1074/mcp.R600010-MCP200 16733262 PMC2790463

[13] Zhang JY Chan EK Peng XX Tan EM. A novel cytoplasmic protein with RNA-binding motifs is an autoantigen in human hepatocellular carcinoma. J Exp Med (1999) 189(7):1101–10. 10.1084/jem.189.7.1101 10190901 PMC2193003

[14] Moscat J Diaz-Meco MT. p62: a versatile multitasker takes on cancer. Trends Biochem Sci (2012) 37(6):230–6. 10.1016/j.tibs.2012.02.008 22424619 PMC3531712

[15] Lu M Nakamura RM Dent ED Zhang JY Nielsen FC Christiansen J Aberrant expression of fetal RNA-binding protein p62 in liver cancer and liver cirrhosis. Am J Pathol (2001) 159(3):945–53. 10.1016/S0002-9440(10)61770-1 11549587 PMC1850441

[16] Duran A Amanchy R Linares JF Joshi J Abu-Baker S Porollo A p62 is a key regulator of nutrient sensing in the mTORC1 pathway. Mol Cell (2011) 44(1):134–46. 10.1016/j.molcel.2011.06.038 21981924 PMC3190169

